# MicroRNA expression profiling defines the impact of electronic cigarettes on human airway epithelial cells

**DOI:** 10.1038/s41598-017-01167-8

**Published:** 2017-04-24

**Authors:** Siva Kumar Solleti, Soumyaroop Bhattacharya, Ausaf Ahmad, Qian Wang, Jared Mereness, Tirumalai Rangasamy, Thomas J. Mariani

**Affiliations:** 1 Division of Neonatology and Program in Pediatric Molecular and Personalized Medicine, Department of Pediatrics, University of Rochester Medical Center, Rochester, NY USA; 2 Department of Biomedical Genetics, University of Rochester Medical Center, Rochester, NY USA; 3 School of Veterinary Medicine, Louisiana State University, Baton Rouge, LA USA; 4 Department of Environmental Medicine, University of Rochester Medical Center, Rochester, NY USA

## Abstract

While all forms of tobacco exposure have negative health effects, the significance of exposure to electronic cigarettes (eCig) is not fully understood. Here, we studied the global effects of eCig on the micro RNA (miRNA) transcriptome in human lung epithelial cells. Primary human bronchial epithelial (NHBE) cells differentiated at air-liquid interface were exposed to eCig liquid. Exposure of NHBE to any eCig liquid resulted in the induction of oxidative stress-response genes including *GCLM, GCLC*, *GPX2*, *NQO1* and *HO-1*. Vaporization of, and/or the presence of nicotine in, eCig liquid was associated with a greater response. We identified 578 miRNAs dysregulated by eCig exposure in NHBE, and 125 miRNA affected by vaporization of eCig liquid. Nicotine containing eCig vapor displayed the most profound effects upon miRNA expression. We selected 8 miRNAs (29A, 140, 126, 374A, 26A-2, 147B, 941 and 589) for further study. We validated increased expression of multiple miRNAs, including miR126, following eCig exposure. We also found significant reduction in the expression of two miR126 target genes, *MYC* and *MRGPRX3*, following exposure. These data demonstrated that eCig exposure has profound effects upon gene expression in human lung epithelial cells, some of which are epigenetically programmed at the level of miRNA regulation.

## Introduction

Electronic nicotine delivery systems (ENDS), popularly known as electronic cigarettes (eCig) are battery-powered aerosolized nicotine delivery products without any combustion or smoke^1^. ENDS are becoming very popular worldwide^2^ as alternatives to smoking^3^. eCigs are considered as a strategy of reducing adverse health effects of tobacco based smoking by providing low-risk nicotine^4^. However, the frequency of eCig and nicotine liquid exposure among young children is increasing rapidly, and with severe outcomes^5^. The safety of eCigs, and the potential harmful health effects of nicotine intake, particularly on the lung are not fully understood. The Food and Drug Administration (FDA) recently announced regulations on eCig use^6^.

eCig liquid is typically composed of a mixture of propylene glycol and vegetable glycerin vehicles with various flavorings, with or without nicotine. Vaporizing e-liquids, at higher temperatures, may result in the generation of known pulmonary toxicants including acrolein, acetaldehyde and formaldehyde^7, 8^. Further, diacetyl, benzaldehyde, 2,3-pentanedione and other chemicals present in some of the flavorings have known adverse respiratory effects^9–13^.

Airway epithelial cells reside at the interface between the host and the environment, and act as the first line of defense against noxious gases, allergens and microorganisms^14–16^. Airway epithelial derived pro-inflammatory molecules are known to play an important role in regulating lung inflammation^17–19^. Further, involvement of respiratory epithelial cells in orchestrating pulmonary immunity and homeostasis is evident^16^. Recent reports suggest that use of eCigs alters innate immunity, neutrophil inflammation and airway cytokines expression while increasing the virulence of colonizing bacteria^20^ increases neutrophil inflammatory response^13^ and virus infection in primary human airway epithelial cells^21^. In addition, exposure of human airway epithelial cells to eCig aerosols induces the secretion of inflammatory cytokines, IL-6 and IL-8^22^.

Exposure of the lung epithelial cells to cigarette smoke induces a variety of effects directly measurable at the cellular and molecular level^23–25^. Increased oxidative stress is a major driving mechanism in the pathophysiology of smoking-related lung diseases such as COPD^18, 26^. The presence of a large amount of free radicals in cigarette smoke^27^ disturbs the redox balance in the lungs, leading to increased oxidative burden^28^. In COPD, antioxidant capacity is reduced and oxidative stress persists even after the cessation of smoking due to the continued production of reactive oxygen species from endogenous sources, contributing to COPD comorbidities such as cardiovascular diseases, metabolic syndrome and lung cancers^26^. Conversely, very little is known about the role of eCigs in oxidative lung damage. A recent report suggested that the oxidative stress inducing capacity of eCigs depends on their flavor additives, with flavors containing sweet or fruit flavors being stronger oxidizers than tobacco flavors^22^. eCig liquids have been shown to profoundly alter the metabolome of bronchial epithelial cells partly similar to cigarette smoke condensate, and the use of antioxidants can partially counteracted this response^10^. Further, soluble components of eCigs, including nicotine, cause loss of lung endothelial barrier function, due to oxidative stress and inflammation^29^. Furthermore, exposure of bronchial epithelial cells to nicotine induces apoptosis and senescence via ROS mediated autophagy-impairment and could act as a potential mechanism for COPD-emphysema pathogenesis^30^.

## Results

### Regulation of oxidative stress (OxS) response genes following electronic cigarette exposure

**Figure 1:**
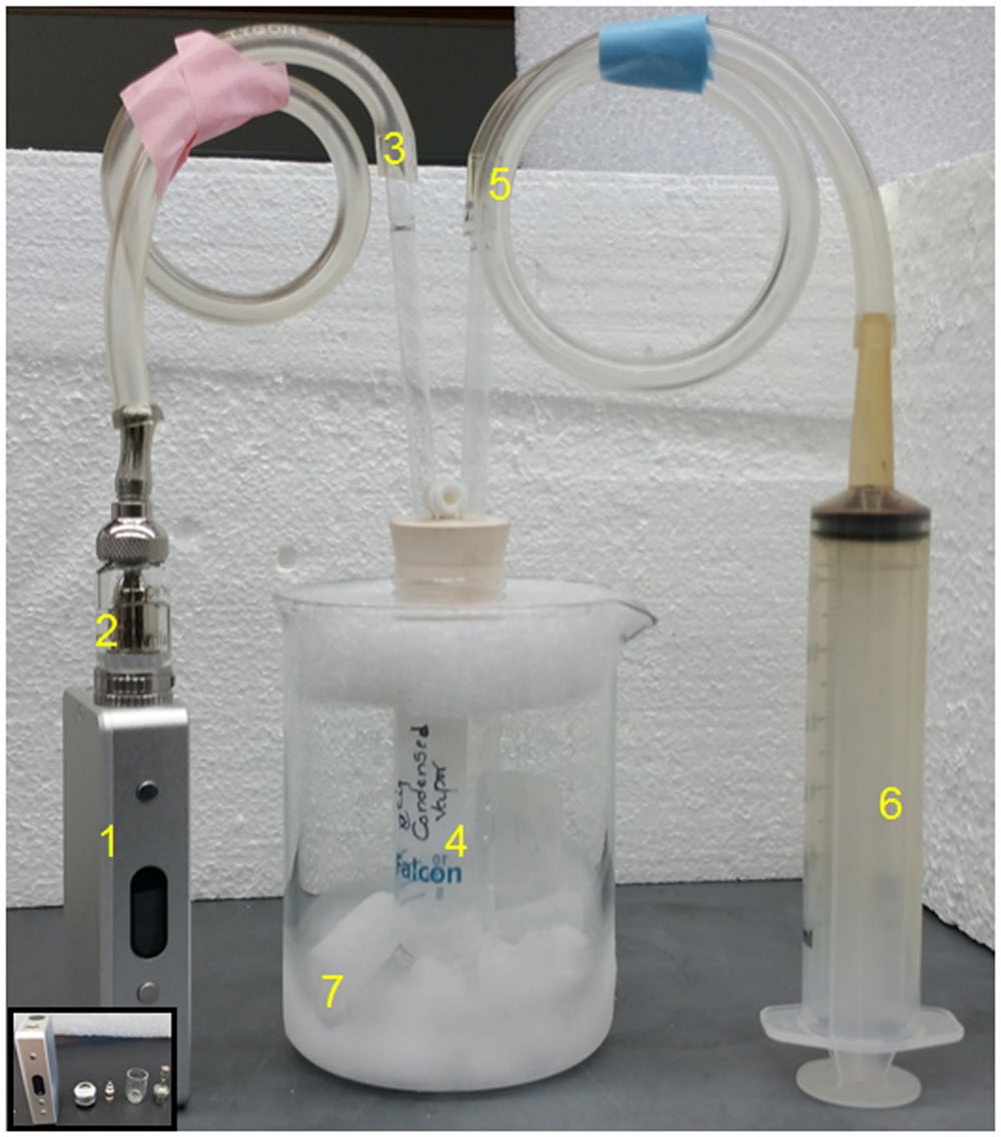
Generation of eCig vapor condensate. eCig liquid was added to the tank system atomizer (1 & inset) and connected to the electronic smoking device (2). Using a custom assembly (3 & 5), 40 ml eCig vapor “puffs” were drawn into a test tube using a 60 ml syringe (4), with each puff lasting for 4 seconds. Vapor spontaneously condensed in the tube (6), which was chilled above a liquid nitrogen/dry ice bath (7).

**Figure 2:**
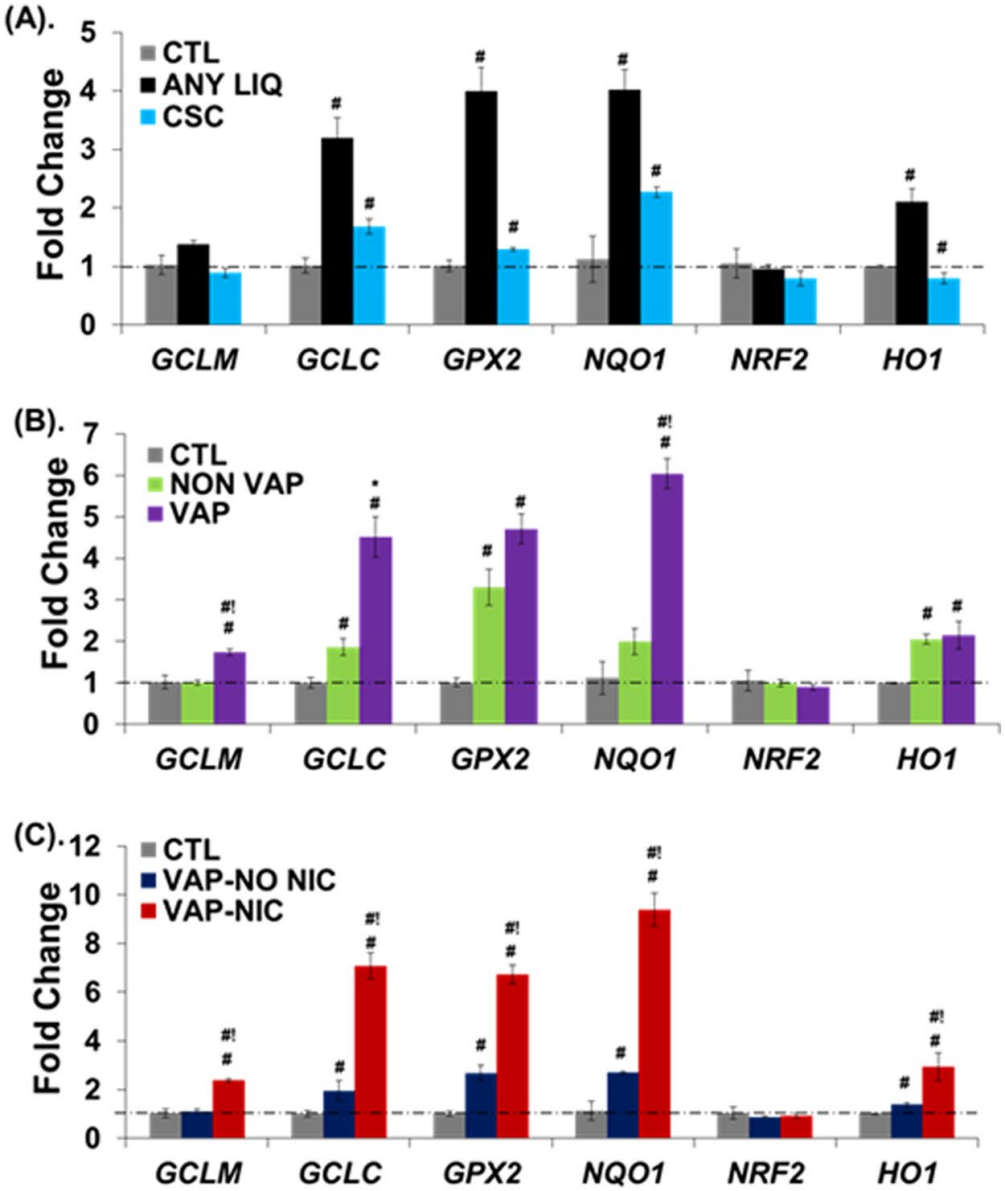
Expression of oxidative stress (OxS) response genes in NHBE cells upon eCig exposure. eCig liquid exposure induces OxS response gene expression in primary human lung epithelial cells. NHBE cells were treated for 2 days with CSC (40 µg), or with 2% non-vaporized (NON VAP) or vaporized and condensed (VAP) eCig liquid, lacking (NO NIC) or containing nicotine (NIC). Following treatment for 48 hours, cells were tested for mRNA expression of OxS genes using qPCR. (**A**) Treatment of cells with any eCig liquid, vaporized or not, with or without nicotine (ANY LIQ) induced the expression of *GCLC*, *GPX2*, *NQO1*, and *HO1*. Attenuated responses were observed for CSC. (**B**) Vaporized and condensed eCig liquid (VAP) displayed a larger response than non-vaporized eCig liquid (NON VAP) on OxS mRNA expression. Treatment of cells with VAP significantly induced the expression of *GCLM*, *GCLC*, *GPX2*, *NQO1* and *HO1* compared to CTL. Expression of *GCLM*, *NQO1* and *GCLC* were further increased in VAP treatment compared to NON VAP treated cells. (**C**) Nicotine containing eCig vapor induced the maximal OxS response among all exposure conditions. VAP with no nicotine (VAP-NO NIC) modestly induced the expression of *GCLC*, *GPX2*, *NQO1* and *HO1* compared to CTL. Conversely, VAP with nicotine (VAP-NIC) induced the expression of *GCLM*, *GCLC*, *GPX2*, *NQO1* and *HO1* compared to CTL. ^#^p < 0.05 (vs CTL); ^#!^p < 0.05 (NON VAP vs VAP or VAP-NO NIC vs VAP-NIC) (MWU). *p < 0.09 (NON VAP vs VAP) (T-test).

Oxidative stress plays an important role in the pathogenesis of COPD^26^. To determine whether exposure to eCig induces oxidative stress in human bronchial epithelial cells, we used qPCR to analyze the expression of key oxidative stress response genes. Treatment of primary human lung epithelial cells (NHBE) with 2% non-vaporized (NON VAP) or vaporized and condensed (VAP) eCig liquid (Fig. 1; Methods), lacking (NO NIC) or containing nicotine (NIC) did not induce any visible signs of cell death (personal observation), as cells appeared healthy and similar to cells treated with medium alone or cigarette smoke condensate (CSC). qPCR analysis indicated that exposure to eCig liquid induced the expression of OxS response genes in primary NHBE cells (Fig. 2A–C). Interestingly, treatment of cells with any eCig liquid (Fig. 2A), vaporized or not, with or without nicotine (ANY LIQ) induced the expression of *GCLC* (glutamate–cysteine ligase, catalytic subunit) (3.1 fold, *p* < 0.05), *GPX2* Glutathione peroxidase 2 (3.9 fold, *p* < 0.05), *NQO1* (NAD(P)H quinone dehydrogenase 1) (3.5 fold, *p* < 0.05), and *HO1* (heme oxygenase (decycling) 1) (2.1 fold, *p* < 0.05). By contrast, treatment with CSC (40 ug/ml) induced only the expression of *GCLC* (1.6 fold, *p* < 0.05), *GPX2* (1.2 fold, *p* < 0.05), *NQO1* (2 fold, *p* < 0.05).

We next specifically tested for the effects of eCig vaporization and nicotine on the OxS responses (Fig. 2B). Vaporized and condense eCig liquid (VAP) treatment significantly increased the expression of *GCLM* (1.7 fold, *p* < 0.05), *NQO1* (3.0 fold, *p* < 0.05) and *GCLC* (2.4 fold, *p* < 0.05) compared to cells treated with non-vaporized eCig liquid (NON VAP). Intriguingly, nicotine containing eCig vapor induced the maximal OxS response among all exposure conditions (Fig. 2C). When compared to VAP with no nicotine (VAP-NO NIC), VAP with nicotine (VAP-NIC) induced *GCLM* (2.1 fold, *p* < 0.05), *GCLC* (3.6 fold, *p* < 0.05), *GPX2* (2.5 fold, *p* < 0.05), *NQO1* (3.4 fold, *p* < 0.05) and *HO1* (2.1 fold, *p* < 0.05).

### Genome-wide transcriptional analysis of microRNA expression in eCig treated NHBE

**Figure 3:**
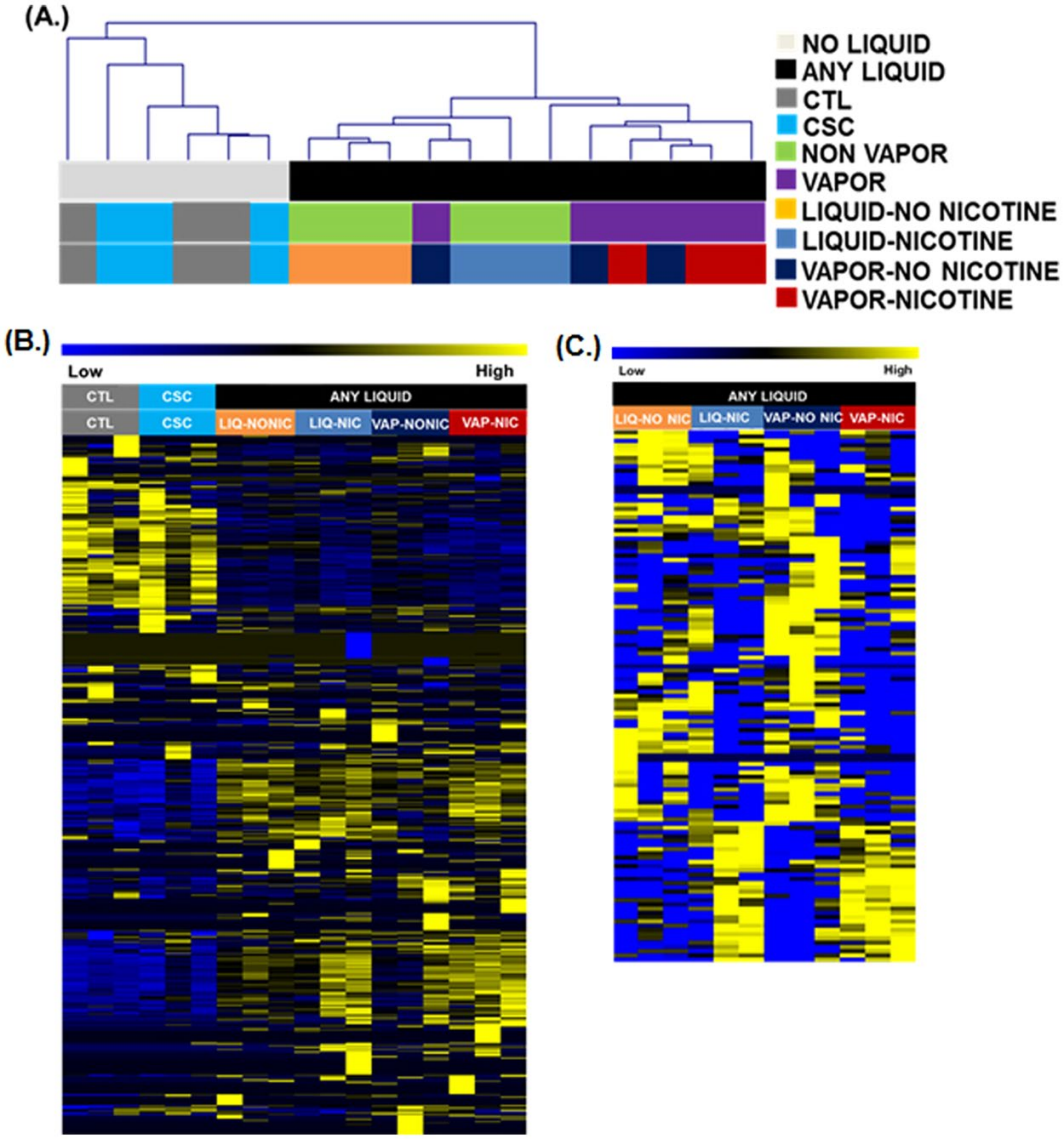
Genome-wide transcriptional analysis of microRNA expression in eCig treated NHBE. (**A**) Treatment with any eCig liquid has a significant effect on miRNA expression. Shown here is a dendrogram representing hierarchical clustering of samples when comparing expression of all 2541 miRNAs. The clusters were generated using Euclidean distance and average linkage clustering. Samples completely segregated based upon treatment with any eCig liquid (black bar). Individual treatment groups were untreated control (CTL), cigarette smoke condensate (CSC), non-vaporized liquid without (LIQ-NONIC) or with (LIQ-NIC) nicotine, and vaporized/condensed liquid without (VAP-NONIC) or with (VAP-NIC) nicotine. (**B**) SAM-Seq identified 578 miRNA significantly differentially expressed between samples with any eCig liquid treatment (n = 12), when compared to controls (n = 3). Shown are normalized expression levels for these 578 selected miRNA. Rows represent miRNA and columns represent samples. Blue indicates low expression, while yellow indicates high expression. (**C**) SAM-Seq identified 125 microRNA as significantly differentially expressed between cells treated with vaporized liquid (n = 6) compared to non-vaporized liquid (n = 6). Shown are normalized expression levels for these 125 selected miRNA. Rows represent miRNA and columns represent samples. Blue indicates low expression, while yellow indicates high expression.

We were particularly interested in exploring epigenetic regulatory mechanisms induced by eCig exposure. Therefore, we transcriptionally profiled 2541 miRNA from NHBE which were either treated with non-vaporized liquid without (LIQ-NONIC), with nicotine (LIQ-NIC), vaporized/condensed liquid without (VAP-NONIC) or with nicotine (VAP-NIC), cigarette smoke condensate (CSC), or untreated control cells (CTL). Hierarchical cluster analysis of the data set indicated a clear segregation among cells treated with any eCig liquid as compared to other groups (Fig. 3A). Similar clusters were observed using non-parametric bootstrap analysis.

SAM-Seq analysis (at median FDR = 0) identified 578 miRNA significantly differentially expressed between samples with any eCig liquid treatment (n = 12), when compared to untreated controls (n = 3) as listed in Supplemental Table 1. The expression profiles for these miRNAs is presented tin Fig. 3B. SAM-Seq (at median FDR = 0) identified 125 microRNAs as significantly differentially expressed between cells treated with any vaporized liquid (n = 6) compared to any non-vaporized liquid (n = 6) as listed in Supplemental Table 2. The expression profiles for these miRNAs is presented in Fig. 3C.

### miRNA-mRNA regulation by electronic cigarette exposure

**Figure 4:**
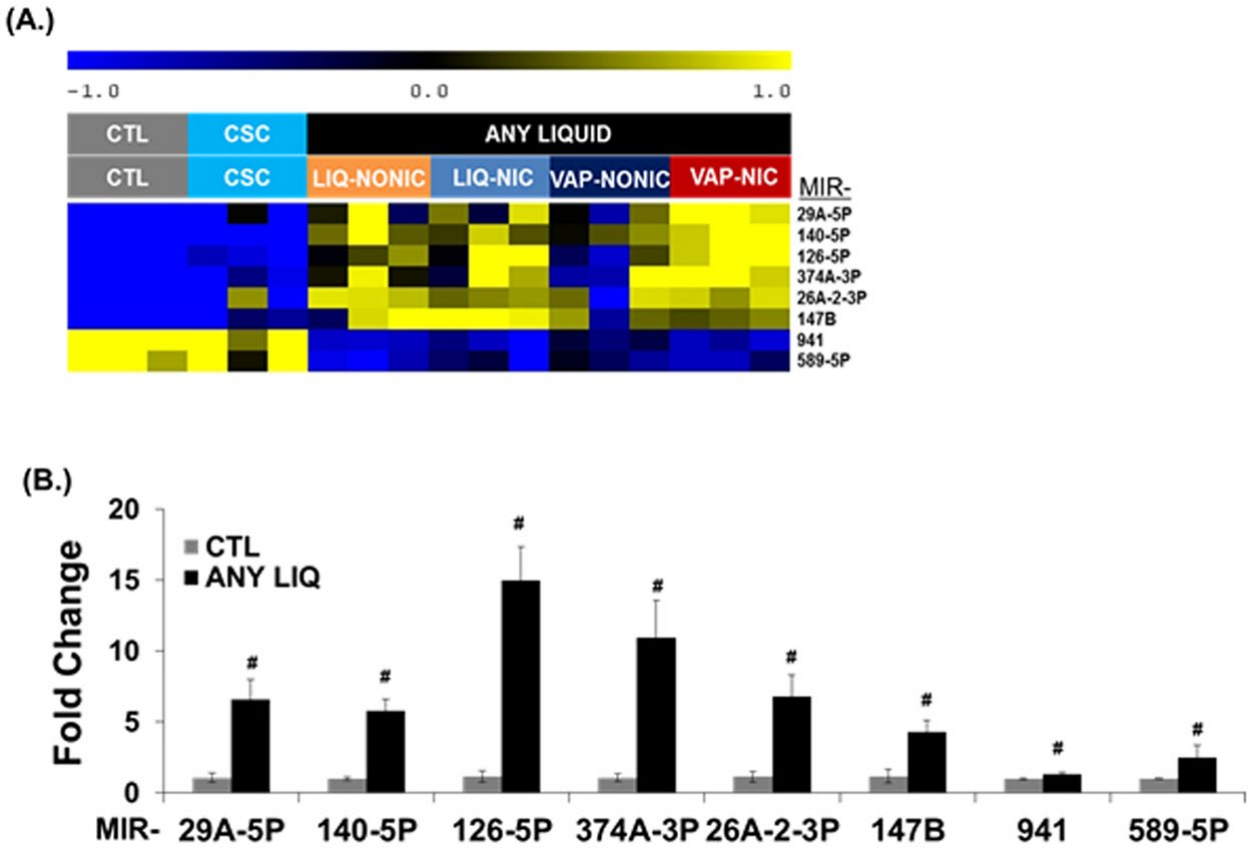
qPCR validation of miRNA expression in eCig exposed human lung epithelial cells (**A**). Differential expression of selected miRNAs. Validation was attempted on eight miRNAs chosen based on their magnitude of change and biological interest. Shown are normalized expression levels for these 8 selected miRNA. Rows represent miRNA and columns represent samples. Blue indicates low expression, while yellow indicates high expression. Individual treatment groups were untreated control (CTL), cigarette smoke condensate (CSC), non-vaporized liquid without (LIQ-NONIC) or with (LIQ-NIC) nicotine, and vaporized/condensed liquid without (VAP-NONIC) or with (VAP-NIC) nicotine. (**B**) Validation of microRNA expression by qPCR. We used qPCR to confirm expression patterns predicted by RNA-Seq analysis for 8 miRNA (MIR29A-5P, MIR140-5P, MIR126P, MIR374A-3P, MIR26A-2-3P and MIR147B, MIR941 and MIR589-5P) that were induced following treatment of any eCig liquid (ANY LIQUID). We successfully validated differences in expression for 6 of these 8 miRNA. ^#^ *p* < 0.05; ^#!^ *p* < 0.056 (MWU) (CTL vs any ANY LIQ).

Based upon the magnitude of response, and biological interest, we attempted to validate differential expression for eight miRNAs predicted to be significantly affected by treatment with any eCig liquid (Fig. 4A). Similar to increased expression predicted by RNA-Seq analysis, we found a significant increase in the expression of MIR26A-2-3P (5.9 fold, p < 0.05), MIR126-5P (12.6 fold, p < 0.05), MIR140-5P (5.6 fold, p < 0.05), MIR29A-5P (6 fold, p < 0.05), MIR374A-3P (10.2 fold, p < 0.05) and MIR147B (3.6 fold, p < 0.05) by qPCR (Fig. 4B). Conversely, expression of MIR941 (50%, p < 0.05) and MIR589-5P (69%, p < 0.05), which were predicted by RNA-Seq analysis to be down regulated, were not validated by qPCR.

**Figure 5:**
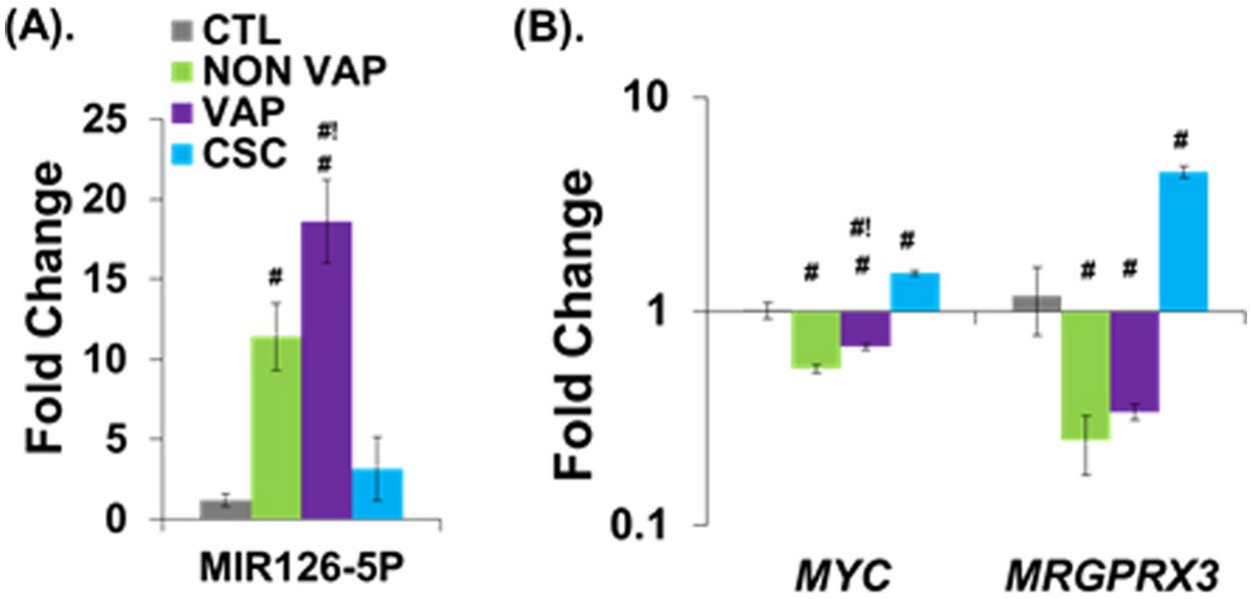
Validation of expression for MIR126-5P and its target mRNAs in eCig treated human lung epithelial cells. Validation of MIR126-5P induction, and suppression of its target mRNAs by eCig liquid. Shown are fold differences in expression (log scale) for each gene upon eCig exposure. (**A**) MIR126-5P is highly induced by eCig liquid, particularly following vaporization. Increased expression of MIR126-5P upon CSC treatment did not reach significance. (**B**) Two MIR126-5P–specific target genes, *MYC* and *GPCR*, display reduced expression following treatment with e-cg liquid. Increased expression of MIR126-5P following treatment with eCig liquid resulted in decreased expression of *MYC* (46% in NON VAP, 31% in VAP) and *MRGPRX3* (75% in NON VAP, 66% in VAP), as compared to CTL cells. ^#^p < 0.05 (MWU) (vs CTL); ^#!^p < 0.05 (NON VAP vs VAP) (MWU).

Based upon these data, we chose to further explore the induction of MIR126-5P, and test whether this results in inhibition of its target genes in response to eCig treatment. MIR126-5P is significantly induced by eCig liquid, particularly following vaporization (Fig. 5A). VAP exposure increased MIR126-5P expression (1.6 fold, p < 0.05) as compared to NON VAP exposure (15.6 fold in VAP vs 9.6 fold in NON VAP, p < 0.05). A modest increase in expression of MIR126-5P noted upon CSC treatment (2.6 fold) did not reach statistical significance.

Next, we tested the expression levels of two MIR126-5P–specific target genes, *MYC* ^31^ and *MRGPRX3* ^32^ (Fig. 5B). Significant increases in MIR126-5P expression following NON VAP or VAP exposure was associated with significantly decreased expression of *MYC* (46% in NON VAP, 31% in VAP) and *MRGPRX3* (75% in NON VAP, 66% in VAP), as compared to CTL cells. Conversely, in CSC treated cells, expression of *MYC* (1.49 fold, p < 0.05) and *MRGPRX3* (3.7 fold, p < 0.05) paralleled the MIR126-5P expression.

## Discussion

MiRNAs are small RNA molecules (21–25 nucleotides long) that regulate gene expression post-transcriptionally, either by mRNA degradation or inhibition of translation or both^33^. MiRNAs are predicted to regulate approximately 60% of protein-coding genes in human^34^. MiRNAs have been recognized as modulators of smoking-induced gene expression changes in human airway epithelium^35^, and dysregulation of miRNA expression are associated with many pulmonary diseases including COPD^35, 36^. Electronic cigarettes have become very popular in recent years, with an estimated 13 million people using globally^2, 13^. ECigs may help reduce tobacco smoking^3, 4, 37^, however the safety of eCigs has yet to be fully appreciated^10, 13, 38, 39^. In the present study we demonstrate that exposure to eCig liquid regulates oxidative stress response genes and numerous miRNAs in human lung cells.

Transcriptional profiling for the assessment of global changes in expression of coding and non-coding RNA species associated with cigarette smoke exposure, *in vitro* or *in vivo*, have been reported^35, 40–45^. Similar analyses of the effects of eCig exposure could help to assess the potential health effects^46^. Transcriptome analysis has identified gene expression profiles in human bronchial epithelial cells upon eCig vapor and mainstream-smoke from tobacco cigarette exposure^47^. Further, transcriptional profiling for immune and inflammatory-response genes in nasal epithelial cells from eCig users has been reported^48^ recently. However, no comprehensive analysis of eCig effects upon lung epithelial cell miRNA expression been previously reported. Our results indicate that eCig exposure induces the expression of oxidative stress response genes, and causes dysregulation of numerous miRNAs in human bronchial epithelial cells *in vitro*.

*In vitro* and *in vivo* studies have shown that miRNAs play a crucial role in cellular response to stress as well as cell growth and death^35, 36, 49, 50^. The airway epithelial miRNA transcriptome changes in response to cigarette smoke exposure^35, 49^, and miRNAs have been implicated in COPD pathology^35, 49, 51^. A main goal of this study was to determine the global effects of eCig exposure upon miRNA expression, and test whether miRNAs alter expression of their target mRNA in eCig-exposed lung epithelial cells. We have identified 578 miRNAs as differentially expressed with eCig treatment. Interestingly, vaporization of eCig liquid resulted in dysregulation of 125 miRNA compared to non-vaporized liquid. While this is the first study to report the miRNA transcriptome profiling in response to eCigs, two recent studies describe global changes in mRNA expression under similar conditions^47, 48^.

Among the eight selected miRNAs, we focused our interest upon MIR126, which is located in chromosome 9q34.3 within the host gene encoding for epidermal growth factor like-7 (*EGFL-7*)^52, 53^. It is highly expressed within highly vascularized adult tissues like the lung, heart, and kidney^54, 55^. Its expression is decreased in lung tumor and appears to be regulated epigenetically by demethylating agents^56, 57^. MIR126 has been shown to regulate various biological processes including inhibition of invasion in non-small cell lung carcinoma^52^, to affect mitochondrial energy metabolism resulting in malignant mesothelioma tumor suppression^58^, and to regulate leucocyte adhesion and transmigration across the endothelium^59^. Soluble components of eCig vapor, including nicotine, have been reported to cause loss of lung endothelial barrier function, associated with oxidative stress and inflammation^29^. We found increased expression of MIR126-5P in all conditions tested, with the greatest effect being in cells exposed to vaporized eCig liquid. Importantly, increased expression of MIR126-5P was associated with reduced expression of its target genes, *MYC* and *MRGPRX3*.

Oxidative stress has been recognized as a major driving mechanism in the pathophysiology of COPD^18, 26, 60^. In COPD, there is an imbalance between antioxidant capacity and oxidative stress due to excessive generation of reactive oxygen species (ROS)^26^. In the present study we show that eCigs are potent inducers of OxS response genes in human bronchial epithelial cells, particularly those that contain nicotine. Similar to our findings, recent reports also suggest that eCigs induce oxidative stress, depending on the presence of flavor additives or nicotine^22, 61^. ECig aerosols have also been shown to contain oxidants and copper nanoparticles, inducing mitochondrial stress evident by elevated levels of mitochondrial ROS from human lung fibroblasts^22, 62^.

We specifically found increased expression of genes (*GCLC* and *GCLM*) that catalyze the production of the cellular endogenous antioxidant glutathione (GSH). Of all antioxidants, higher concentrations of GSH have been shown to be present in the lungs and these levels are even further increased in smokers^63^. Likewise, we found eCigs increase the expression of glutathione peroxidases (*GPX*), the primary antioxidant enzymes that scavenge and detoxify hydrogen peroxide and organic hydroperoxides, which are also increased in the lungs of cigarette smoke-exposed mice^64^. We have also found increased expression of *NQO1* and *HO1*. *NQO1* is a flavoprotein, which exerts either antioxidant or pro-oxidant properties depending on the quinone substrate. *HO1*, the inducible isoform of heme oxygenase, is a cytoprotective enzyme that plays a critical role in the defense against oxidative and inflammatory insults in the lung^65^. The basic leucine zipper transcription factor, *Nrf2* plays an important role in the transcriptional regulation of oxidative stress response genes. However, eCigs did not induce the expression of *Nrf2* in bronchial epithelial cells. Nuclear translocation of *Nrf2*, not necessarily expression levels, have been shown to be important in the transcriptional induction of antioxidant and phase II detoxification genes in different cells^25^.

The relationship between eCig-induced oxidative stress and miR dysregulation is currently unclear. In the current study, we used oxidative stress gene expression responses as a measure (positive control) for previously reported effects of eCig liquid, and further explored the effects of vaporization and nicotine content on these responses. We were also interested in potential “epigenetic” effects of eCig liquid exposure in lung epithelial cells. Therefore, we surveyed miRNA dysregulaiton by eCig liquid at the genome-wide level. We queried multiple databases (mirPath^66^, Ingenuity Pathway Analysis) for functional analysis of the differentially expressed miRNAs we identified, in order to determine if they (as a set) had pre-existing relationships with regulating oxidative stress response genes. We did not find any associations; most likely due to lack of sufficient annotations for function of miRs in existing databases.

In summary, we have profiled global miRNA expression in the bronchial epithelium exposed to electronic cigarettes. Our findings suggest that exposure to eCigs induces oxidative stress response gene expression and causes dysregulation of many miRNAs in bronchial epithelial cells. We have further demonstrated anti correlation between MIR126-5P and its target genes, *MYC* and *MRGPRX3*. MicroRNA profiles observed from this study might therefore serve as biomarkers for defining eCig exposure, as well as their potential pathophysiological effects.

## Materials and Methods

### Materials

Absolute RNA miRNA kit (Agilent, #400814, Stratagene, La Jolla, CA), normal human bronchial epithelial cells (NHBE; Lonza, Mapleton, IL), bronchial epithelial basal medium (BEBM, Lonza), Dulbecco’s modified Eagle medium (DMEM), PneumaCult-ALI medium (Stemcell Technologies, Vancouver, Canada), electronic smoking device (IPV Technology, Shenzhen, China), eCig liquid without or with nicotine (0% or 2.4% nicotine, V2, Miami, FL), Nautilus Mini tank system (Nautilus Mini adjustable airflow tank system, Aspire, Kent, WA), Taqman MicroRNA reverse transcription kit (Applied Biosystems, Foster City, CA), iScript cDNA Synthesis Kit, SYBR Green chemistry (Applied Biosystems).

### Cell Culture

NHBE cells were cultured as previously described, with modifications^19^, and used between passages 2 and 4. Briefly, NHBE cells were expanded in bronchial epithelial basal medium (BEBM, Lonza) containing bovine pituitary extract, hydrocortisone, human recombinant epidermal growth factor (25 ng/mL), epinephrine, insulin, triiodothyronine, transferrin, gentamicin, Amphotericin B, retinoic acid, and BSA. Cells were transferred to rat tail collagen-coated Transwell inserts (12 well PET membrane, 0.4 µm pore size, 12 mm diameter) for Air-Liquid Interface culture (ALI). When the cells in the transwell plate reached confluence (10–12 days), with appropriate resistance (200–300 Ohms/cm^2^), cells were transferred to ALI by removing the apical medium, and cultured for additional 14 days. For growth of cells on transwell plates, the basal medium was modified to a 1:1 mixture of BEBM/Dulbecco modified Eagle medium (DMEM) with high glucose containing the same supplements, except with a lower concentration of human recombinant epidermal growth factor (0.5 ng/mL). Upon transition to ALI, cells were maintained using PneumaCult-ALI medium (Stemcell technologies, Canada) containing PneumaCult-ALI maintenance supplements and hydrocortisone, according to manufacturer’s instructions. The medium was replaced every 48 hrs.

### Preparation of Electronic Cigarette Condensate and Treatment

Figure 1 presents the apparatus used to prepare electronic cigarette condensate. Briefly, eCig liquid, without or with nicotine (0% or 2.4% w/v nicotine, respectively), was added to a Nautilus Mini tank system with 1.8 ohm BVC atomizer, and connected to a charged electronic smoking device. Using a custom tube assembly, 40 ml “puffs” were drawn with a 60 ml syringe, with each puff lasting for 4 seconds. The puffs were drawn at 7.5 W output power settings of the electronic smoking device. The eCig vapor condensed in a 50 ml falcon tube chilled above a liquid nitrogen/dry ice bath. Using this assembly, 1000 ml eCig vapor yielded approximately 20 µl of condensate. Freshly prepared eCig condensates were used for every experiment. Cells were treated with eCig liquid, vaporized and condensed eCig liquid (2%) or cigarette smoke condensate^19^ (Murthy pharmaceuticals, City, State), baso-laterally for 48 hrs. Cells treated with medium alone, served as control. This concentration was selected based upon preparatory studies indicating graded dose-response effects of concentrations between 0.1–2.0% upon oxidative stress response gene expression.

### RNA Isolation and Reverse Transcription and Real-Time Quantitative RT-PCR (qPCR)

DNA free-total RNA was isolated from cultured cells, using the Absolute RNA miRNA kit. For mRNA analysiss, total RNAs were reverse-transcribed using the iScript cDNA Synthesis Kit. qPCR analysis was performed using using SYBR Green chemistry. Quantitative real-time PCR Gene expression levels were calculated relative to *Ppia* (cyclophilin A) using the ddCT method as we have previously described^19, 67^. Our choice of *Ppia* was based upon results of our previous analysis of gene expression in lung tissues and cells, from different age groups and conditions, where we found Cyclophillin A (PPIA) to be highly stable across all conditions. Consistent with our observations, PPIA was found to be a better candidate housekeeping gene than GAPDH and ACTB, when considering variability of expression^68, 69^. In fact, in prior human studies^42^ we have found that PPIA alone provided equivalent sensitivity and resolution compared to multi-gene normalization approach GeNorm^70^. Primer sequences were selected from PrimerBank (http://pga.mgh.harvard.edu/primerbank/). For miRNA analysis, total RNAs were reverse transcribed using Taqman miRNA reverse transcription kit with minor modifications. For a 15 µl reaction, total RNA (100ng) was combined with RT master mix (0.2 µl dNTP, 0.19 µl RNase-Inhibitor, 1 µl microRNA specific primer, 1 µl multiscribe RT enzyme and 1.5 µl buffer). qPCR analysis was performed using microRNA specific primers supplied with Taqman MicroRNA assay. U6 snRNA was used as normalization control. Small non-coding RNA U6 is a widely used normalization control for microRNAs, has been reported to have stable expression, and was used at the recommendation of the manufacturer of the TaqMan-based miRNA quantitation assay reagents (Thermo Fisher Scientific, Foster City, CA). Gene expression levels were calculated using the ddCT method as we have previously described^19, 67^. Statistical analysis for differences in gene expression by qPCR was performed using either parametric (student’s T) or non-parametric (Mann-Whitney U) tests of the mean.

### microRNA sequencing

Sequencing was performed on cDNA libraries generated using 1000 ng of total RNA from each sample. cDNA quantity was determined with the Qubit Flourometer (Life Technologies, Grand Island, NY) and quality was assessed using the Agilent Bioanalyzer 2100 (Santa Clara, CA). Library construction was performed using the TruSeq Small RNA Sample Preparation kit (Illumina, San Diego, CA). Libraries were quantified with the Qubit Flourometer (Life Technologies, Grand Island, NY) and quality was assessed using the Agilent Tape Station (Santa Clara, CA). Libraries were sequenced (single end reads) on the Illumina HiSeq2500 (Illumina, San Diego, CA) to generate 50 million reads/sample. MiRNA reads were aligned and mapped using miRGE^71^, and summarized at the raw reads level using the following components: *Cutadapt* for sequence read filtering and adapter trimming, and *Bowtie* for sequence read alignment to known mature human microRNA sequences. Raw reads obtained from each alignment algorithm were normalized using reads per million (RPM) bases. Hierarchical clustering, using Euclidean distance and average linkage, with non-parametric bootstrap was used to look for underlying unanticipated association among samples^72^. Differential gene expression was assessed by SAM-Seq^73, 74^ which implements an FDR based approach for correction for multiple testing^75^.

## Electronic supplementary material

Supplemental Tables.
