# Supplementary material for: MicroRNA expression profiling defines the impact of electronic cigarettes on human airway epithelial cells
Source: Sci Rep. 2017 Apr 24;7:1081. doi: 10.1038/s41598-017-01167-8 (PMC5430826; doi:10.1038/s41598-017-01167-8)
Supplement: Supplementary file 1 — Supplemental Tables. [file 41598_2017_1167_MOESM1_ESM.pdf]

SUPPLEMENTAL INFORMATION

MicroRNA expression profiling defines the impact of electronic cigarettes on human airway epithelial cells.

Siva Kumar Solleti[1], Soumyaroop Bhattacharya[1], Ausaf Ahmad[1], Qian Wang[1], Jared Mereness[1,2,] Tirumalai Rangasamy[3], Thomas J. Mariani[1,2,,4]*

[1]Division of Neonatology and Program in Pediatric Molecular and Personalized Medicine, Department of Pediatrics, [2]Department of Biomedical Genetics, and [4]Department of Environmental Medicine, University of Rochester Medical Center, Rochester NY, and [3]School of Veterinary Medicine, Louisiana State University, Baton Rouge, LA

* Corresponding Author

Address for Correspondence:
Thomas J Mariani, PhD
Division of Neonatology and
Pediatric Molecular and Personalized Medicine Program
University of Rochester Medical Center
601 Elmwood Ave, Box 850
Rochester, NY 14642, USA.
Phone: 585-276-4616; Fax: 585-276-2643;
E-mail: Tom_Mariani@urmc.rochester.edu.

Running Title: electronic cigarette miRNA profile

Supplemental Table 1. SAM-Seq analysis (at median FDR=0) identified 578 miRNA significantly differentially expressed between samples with any eCig liquid treatment (n=12), when compared to untreated controls (n=3).

miRNA	Observed SAM score (d)
hsa-miR-421	-8.84
hsa-miR-23a-3p	-8.75
hsa-miR-186-5p	-8.47
hsa-miR-140-5p	-7.40
hsa-let-7f-5p	-6.61
hsa-miR-28-5p	-6.56
hsa-miR-130a-3p	-6.55
hsa-miR-98-5p	-6.42
hsa-miR-151a-3p	-6.26
hsa-miR-182-5p	-6.11
hsa-miR-26b-5p	-6.08
hsa-miR-152-3p	-6.07
hsa-miR-100-5p	-5.87
hsa-miR-215-5p/192-5p	-5.78
hsa-miR-548o-3p	-5.72
hsa-miR-944	-5.62
hsa-miR-365a-3p	-5.58
hsa-miR-99a-5p	-5.58
hsa-miR-147b	-5.57
hsa-miR-34a-5p	-5.54
hsa-miR-30b-5p/30c-5p	-5.42
hsa-miR-454-3p	-5.38
hsa-miR-21-5p	-5.31
hsa-miR-30e-3p	-5.29
hsa-miR-25-3p	-5.29
hsa-miR-223-3p	-5.16
hsa-miR-194-5p	-5.13
hsa-miR-30e-5p	-5.13
hsa-miR-26a-2-3p	-5.12
hsa-let-7a-5p/7c-5p	-5.12
hsa-miR-148a-3p	-5.09
hsa-miR-34a-3p	-5.01
hsa-miR-151b/151a-5p	-4.83
hsa-miR-16-5p	-4.82
hsa-miR-200a-3p	-4.82
hsa-let-7d-5p	-4.81
hsa-miR-455-5p	-4.78
hsa-miR-1299	-4.73
hsa-miR-335-5p	-4.71

hsa-miR-24-2-5p	-4.68
hsa-miR-185-5p	-4.64
hsa-miR-22-3p	-4.59
hsa-miR-10a-5p	-4.58
hsa-miR-301a-3p	-4.52
hsa-miR-629-5p	-4.47
hsa-miR-15a-5p	-4.46
hsa-miR-128-3p	-4.40
hsa-miR-429	-4.37
hsa-miR-452-5p	-4.35
hsa-miR-708-3p	-4.26
hsa-miR-582-3p	-4.25
hsa-miR-101-3p	-4.09
hsa-miR-330-3p	-4.07
hsa-miR-135b-5p	-4.07
hsa-miR-660-5p	-4.05
hsa-miR-193a-3p	-4.02
hsa-let-7a-3p	-3.95
hsa-miR-628-3p	-3.95
hsa-miR-141-5p	-3.88
hsa-miR-374a-5p	-3.87
hsa-miR-301b-3p	-3.85
hsa-miR-96-5p	-3.83
hsa-miR-141-3p	-3.81
hsa-miR-26b-3p	-3.76
hsa-miR-29a-5p	-3.72
hsa-miR-374a-3p	-3.71
hsa-miR-106b-5p	-3.65
hsa-miR-126-5p	-3.65
hsa-let-7b-3p	-3.64
hsa-miR-181a-3p	-3.62
hsa-miR-19b-3p	-3.62
hsa-miR-628-5p	-3.53
hsa-miR-1285-3p	-3.50
hsa-miR-32-5p	-3.48
hsa-miR-664a-5p	-3.45
hsa-miR-17-5p/106a-5p	-3.44
hsa-miR-450b-5p	-3.43
hsa-miR-20a-5p	-3.38
hsa-miR-10b-5p	-3.37
hsa-miR-205-3p	-3.36
hsa-let-7g-5p	-3.31
hsa-miR-548av-5p/548k	-3.28
hsa-miR-190a-5p	-3.25
hsa-miR-450a-5p	-3.25
hsa-miR-126-3p	-3.24

hsa-miR-9-5p	-3.08
hsa-miR-4775	-3.05
hsa-miR-18a-5p	-2.98
hsa-miR-30a-5p	-2.90
hsa-miR-107/103a-3p/103b	-2.76
hsa-miR-582-5p	-2.74
hsa-miR-27a-3p/27b-3p	-2.72
hsa-miR-224-5p	-2.72
hsa-miR-200c-5p	-2.69
hsa-miR-22-5p	-2.63
hsa-miR-340-3p	-2.58
hsa-miR-548e-3p	-2.58
hsa-miR-135b-3p	-2.57
hsa-miR-625-3p	-2.57
hsa-miR-590-3p	-2.50
hsa-miR-19a-3p	-2.49
hsa-miR-340-5p	-2.46
hsa-miR-146b-5p	-2.46
hsa-miR-143-3p	-2.45
hsa-miR-561-5p	-2.45
hsa-miR-4482-3p	-2.43
hsa-miR-4677-3p	-2.42
hsa-miR-409-3p	-2.39
hsa-miR-3607-3p	-2.39
hsa-miR-29c-3p	-2.38
hsa-miR-934	-2.36
hsa-miR-598-3p	-2.30
hsa-miR-24-1-5p	-2.28
hsa-miR-411-5p	-2.25
hsa-miR-3912-3p	-2.25
hsa-miR-135a-5p	-2.24
hsa-miR-16-2-3p	-2.23
hsa-miR-23c	-2.23
hsa-miR-4758-3p	-2.16
hsa-miR-3613-5p	-2.14
hsa-miR-26a-1-3p	-2.10
hsa-miR-424-5p	-2.09
hsa-miR-542-3p	-2.08
hsa-miR-34b-5p	-2.06
hsa-miR-30d-3p	-2.06
hsa-miR-1307-5p	-2.05
hsa-miR-29b-3p	-2.04
hsa-miR-152-5p	-2.03
hsa-miR-203a-5p	-2.01
hsa-miR-20b-5p	-2.00
hsa-miR-6716-3p	-1.97

hsa-miR-664a-3p	-1.93
hsa-miR-203a-3p	-1.91
hsa-miR-651-5p	-1.87
hsa-miR-2355-5p	-1.86
hsa-miR-181c-5p	-1.80
hsa-miR-199a-3p	-1.78
hsa-miR-7-5p	-1.78
hsa-miR-27b-5p	-1.75
hsa-miR-7705	-1.74
hsa-miR-17-3p	-1.73
hsa-miR-4662a-5p	-1.73
hsa-miR-381-3p	-1.72
hsa-miR-101-5p	-1.68
hsa-miR-137	-1.68
hsa-miR-148b-5p	-1.62
hsa-miR-33a-5p	-1.62
hsa-miR-361-5p	-1.61
hsa-miR-7-1-3p	-1.59
hsa-let-7f-1-3p	-1.59
hsa-miR-454-5p	-1.59
hsa-miR-6744-5p	-1.55
hsa-miR-183-3p	-1.53
hsa-miR-627-5p	-1.53
hsa-miR-15b-3p	-1.52
hsa-miR-3613-3p	-1.50
hsa-miR-4454	-1.49
hsa-miR-335-3p	-1.48
hsa-miR-654-3p	-1.47
hsa-miR-221-3p	-1.47
hsa-miR-548i	-1.47
hsa-miR-3651	-1.44
hsa-miR-1287-5p	-1.44
hsa-miR-200a-5p	-1.44
hsa-miR-4746-5p	-1.43
hsa-miR-374b-3p	-1.43
hsa-miR-3609	-1.42
hsa-miR-130b-3p	-1.39
hsa-miR-34c-5p	-1.39
hsa-miR-146a-5p	-1.37
hsa-miR-129-5p	-1.37
hsa-miR-326	-1.35
hsa-miR-6499-5p	-1.34
hsa-miR-744-3p	-1.34
hsa-miR-6852-5p	-1.33
hsa-miR-3929	-1.33
hsa-miR-23b-5p	-1.32

hsa-miR-548e-5p	-1.31
hsa-miR-6786-3p	-1.31
hsa-miR-1226-3p	-1.30
hsa-miR-6516-5p	-1.29
hsa-miR-410-3p	-1.28
hsa-miR-34b-3p	-1.27
hsa-miR-32-3p	-1.27
hsa-miR-1277-5p	-1.27
hsa-miR-20a-3p	-1.27
hsa-miR-550a-3p	-1.27
hsa-miR-24-3p	-1.26
hsa-miR-199b-5p	-1.25
hsa-miR-181c-3p	-1.25
hsa-miR-23b-3p	-1.22
hsa-miR-6516-3p	-1.22
hsa-miR-3913-5p	-1.22
hsa-miR-365b-3p	-1.22
hsa-miR-576-3p	-1.20
hsa-miR-616-5p	-1.12
hsa-miR-652-5p	-1.11
hsa-miR-203b-3p	-1.11
hsa-miR-33b-5p	-1.10
hsa-miR-4709-3p	-1.10
hsa-miR-103a-2-5p	-1.09
hsa-miR-1229-3p	-1.08
hsa-miR-338-3p	-1.04
hsa-miR-1246	-1.04
hsa-miR-132-5p	-1.04
hsa-miR-129-1-3p	-1.04
hsa-miR-532-5p	-1.02
hsa-miR-2355-3p	-1.02
hsa-let-7c-3p	-1.01
hsa-miR-5001-3p	-1.00
hsa-miR-219a-5p	-0.99
hsa-miR-1248	-0.94
hsa-miR-6511b-3p	-0.94
hsa-miR-6854-5p	-0.93
hsa-miR-182-3p	-0.93
hsa-miR-191-3p	-0.92
hsa-miR-100-3p	-0.92
hsa-miR-143-5p	-0.92
hsa-miR-6810-3p	-0.90
hsa-miR-195-5p	-0.90
hsa-miR-3928-3p	-0.90
hsa-miR-6769b-3p	-0.89

hsa-miR-548au-5p/548c-5p/548o-5p/548am-5p	-0.89
hsa-miR-6837-3p	-0.84
hsa-miR-485-5p	-0.83
hsa-miR-3934-5p	-0.83
hsa-miR-545-5p	-0.81
hsa-miR-4636	-0.80
hsa-miR-449c-3p	-0.80
hsa-miR-497-5p	-0.79
hsa-miR-4423-3p	-0.78
hsa-miR-1295a	-0.77
hsa-miR-6513-5p	-0.75
hsa-miR-188-3p	-0.75
hsa-miR-223-5p	-0.74
hsa-miR-33a-3p	-0.74
hsa-miR-1255a	-0.73
hsa-miR-2277-3p	-0.72
hsa-miR-6735-5p	-0.72
hsa-miR-186-3p	-0.72
hsa-miR-503-5p	-0.72
hsa-miR-3664-3p	-0.72
hsa-miR-6514-5p	-0.72
hsa-miR-668-3p	-0.72
hsa-miR-6724-5p	-0.72
hsa-miR-181b-2-3p	-0.71
hsa-miR-218-5p	-0.71
hsa-miR-1277-3p	-0.71
hsa-miR-30b-3p	-0.71
hsa-miR-362-3p	-0.70
hsa-miR-323a-3p	-0.70
hsa-miR-3177-3p	-0.70
hsa-miR-5000-3p	-0.70
hsa-miR-3157-3p	-0.70
hsa-miR-365a-5p	-0.68
hsa-miR-449a	-0.68
hsa-miR-181b-3p	-0.68
hsa-miR-142-5p	-0.67
hsa-miR-183-5p	-0.67
hsa-let-7g-3p	-0.67
hsa-miR-95-5p	-0.67
hsa-miR-502-5p	-0.66
hsa-miR-6730-5p	-0.66
hsa-miR-425-5p	-0.65
hsa-miR-659-5p	-0.65
hsa-miR-153-3p	-0.63
hsa-miR-590-5p	-0.63

hsa-miR-942-5p	-0.63
hsa-miR-5699-5p	-0.61
hsa-miR-4787-3p	-0.61
hsa-miR-556-5p	-0.61
hsa-miR-5690	-0.56
hsa-miR-6502-5p	-0.56
hsa-miR-10a-3p	-0.56
hsa-miR-499a-5p	-0.56
hsa-miR-3166	-0.55
hsa-miR-6866-5p	-0.55
hsa-miR-4781-3p	-0.53
hsa-miR-200b-3p	-0.52
hsa-miR-4521	-0.52
hsa-miR-193b-5p	-0.51
hsa-miR-1276	-0.50
hsa-miR-548ad-5p	-0.49
hsa-miR-5001-5p	-0.49
hsa-miR-4786-5p	-0.49
hsa-miR-3177-5p	-0.49
hsa-miR-3690	-0.49
hsa-miR-6734-5p	-0.49
hsa-miR-4690-5p	-0.49
hsa-miR-3180-5p	-0.49
hsa-miR-935	-0.49
hsa-miR-6505-5p	-0.49
hsa-miR-7976	-0.49
hsa-miR-194-3p	-0.49
hsa-miR-145-3p	-0.49
hsa-miR-323b-3p	-0.49
hsa-miR-551a	-0.49
hsa-miR-3187-3p	-0.49
hsa-miR-6761-5p	-0.49
hsa-miR-3944-3p	-0.49
hsa-miR-1179	-0.49
hsa-miR-548ap-3p/548t-3p/548aa	-0.49
hsa-miR-509-3p	-0.49
hsa-miR-493-5p	-0.49
hsa-miR-6508-5p	-0.49
hsa-let-7f-2-3p	-0.49
hsa-miR-6772-3p	-0.49
hsa-miR-6784-3p	-0.49
hsa-miR-451a	-0.49
hsa-miR-6886-5p	-0.49
hsa-miR-3182	-0.49
hsa-miR-377-3p	-0.49
hsa-miR-9-3p	-0.49

hsa-miR-4768-5p	-0.49
hsa-miR-4446-3p	-0.49
hsa-miR-136-3p	-0.49
hsa-miR-338-5p	-0.49
hsa-miR-1260a	-0.49
hsa-miR-6730-3p	-0.49
hsa-miR-4708-3p	-0.49
hsa-miR-378i	-0.49
hsa-miR-3174	-0.49
hsa-miR-549a	-0.49
hsa-miR-4797-3p	-0.49
hsa-miR-6892-5p	-0.49
hsa-miR-6721-5p	-0.49
hsa-miR-660-3p	-0.49
hsa-miR-6732-3p	-0.49
hsa-miR-3133	-0.49
hsa-miR-758-3p	-0.49
hsa-miR-939-5p	-0.49
hsa-miR-3664-5p	-0.49
hsa-miR-4673	-0.49
hsa-miR-365b-5p	-0.49
hsa-miR-4685-3p	-0.49
hsa-miR-629-3p	-0.49
hsa-miR-3940-3p	-0.49
hsa-miR-3150b-3p	-0.49
hsa-miR-6741-3p	-0.49
hsa-miR-6874-3p	-0.49
hsa-miR-369-5p	-0.49
hsa-miR-548v	-0.49
hsa-miR-4751	-0.49
hsa-miR-6847-5p	-0.49
hsa-miR-5705	-0.49
hsa-miR-3936	-0.49
hsa-miR-1185-1-3p	-0.49
hsa-miR-6818-5p	-0.49
hsa-miR-4785	-0.49
hsa-miR-3617-5p	-0.49
hsa-miR-4697-3p	-0.49
hsa-miR-3614-5p	-0.49
hsa-miR-548l	-0.49
hsa-miR-6875-5p	-0.49
hsa-miR-760	-0.49
hsa-miR-3064-5p	-0.49
hsa-miR-4520-3p	-0.49
hsa-miR-146a-3p	-0.49
hsa-miR-5696	-0.49

hsa-miR-548ap-5p/548j-5p	-0.49
hsa-miR-636	-0.49
hsa-miR-6868-3p	-0.48
hsa-miR-204-5p	-0.48
hsa-miR-548az-5p	-0.48
hsa-miR-937-3p	0.38
hsa-miR-449b-5p	0.39
hsa-miR-625-5p	0.40
hsa-miR-4326	0.42
hsa-miR-187-3p	0.46
hsa-miR-1185-5p	0.49
hsa-miR-19a-5p	0.49
hsa-miR-5683	0.49
hsa-miR-192-3p	0.49
hsa-miR-548u	0.49
hsa-miR-548ay-3p	0.49
hsa-miR-6743-3p	0.49
hsa-miR-216a-3p	0.49
hsa-miR-4802-3p	0.49
hsa-miR-4473	0.49
hsa-miR-6803-3p	0.49
hsa-miR-3677-3p	0.49
hsa-miR-139-5p	0.49
hsa-miR-1284	0.49
hsa-miR-4687-5p	0.49
hsa-miR-3173-5p	0.49
hsa-miR-675-3p	0.49
hsa-miR-545-3p	0.49
hsa-miR-548n	0.49
hsa-miR-2116-5p	0.49
hsa-miR-579-5p	0.49
hsa-miR-1538	0.49
hsa-miR-494-3p	0.49
hsa-miR-642a-3p	0.49
hsa-miR-5702	0.49
hsa-miR-4707-3p	0.49
hsa-miR-4477b	0.49
hsa-miR-5588-5p	0.49
hsa-miR-6810-5p	0.49
hsa-miR-4654	0.49
hsa-miR-4742-5p	0.52
hsa-miR-550a-5p	0.52
hsa-miR-3605-5p	0.53
hsa-miR-2682-5p	0.54
hsa-miR-641	0.56
hsa-miR-4423-5p	0.57

hsa-miR-150-5p	0.59
hsa-miR-127-3p	0.60
hsa-miR-4640-3p	0.60
hsa-miR-1249-3p	0.62
hsa-miR-29b-2-5p	0.62
hsa-miR-1291	0.63
hsa-miR-1247-5p	0.63
hsa-miR-3127-5p	0.67
hsa-miR-3616-5p	0.68
hsa-miR-5581-3p	0.70
hsa-miR-378a-5p	0.72
hsa-miR-887-5p	0.74
hsa-miR-184	0.76
hsa-miR-181d-5p	0.76
hsa-miR-505-5p	0.78
hsa-miR-140-3p	0.78
hsa-miR-92b-5p	0.80
hsa-miR-5010-3p	0.81
hsa-miR-576-5p	0.81
hsa-miR-769-3p	0.82
hsa-miR-4286	0.84
hsa-miR-1266-5p	0.87
hsa-miR-375	0.93
hsa-miR-345-5p	0.94
hsa-miR-222-5p	0.94
hsa-miR-1268b	0.98
hsa-miR-33b-3p	0.99
hsa-miR-374b-5p	1.09
hsa-miR-4728-3p	1.11
hsa-miR-212-5p	1.11
hsa-miR-2116-3p	1.12
hsa-miR-191-5p	1.12
hsa-miR-362-5p	1.16
hsa-miR-940	1.17
hsa-let-7i-5p	1.18
hsa-miR-3158-3p	1.21
hsa-miR-181a-2-3p	1.21
hsa-miR-29c-5p	1.25
hsa-miR-1260b	1.26
hsa-miR-210-3p	1.27
hsa-miR-542-5p	1.27
hsa-miR-7974	1.28
hsa-miR-221-5p	1.29
hsa-miR-4645-3p	1.30
hsa-miR-145-5p	1.30
hsa-miR-4449	1.33

hsa-miR-193a-5p	1.35
hsa-miR-449b-3p	1.39
hsa-miR-1270	1.49
hsa-miR-664b-3p	1.50
hsa-miR-1271-5p	1.54
hsa-miR-3909	1.55
hsa-miR-92b-3p	1.56
hsa-miR-23a-5p	1.59
hsa-miR-1292-5p	1.59
hsa-miR-185-3p	1.60
hsa-let-7b-5p	1.69
hsa-miR-6814-3p	1.70
hsa-miR-3620-3p	1.72
hsa-miR-342-3p	1.77
hsa-miR-504-5p	1.91
hsa-miR-449c-5p	1.93
hsa-miR-664b-5p	1.95
hsa-miR-6859-5p	2.01
hsa-miR-3176	2.03
hsa-miR-1247-3p	2.10
hsa-miR-1273d	2.11
hsa-miR-4709-5p	2.14
hsa-miR-671-5p	2.15
hsa-miR-548p	2.18
hsa-miR-373-3p	2.18
hsa-miR-378a-3p/378c/378d/378e	2.22
hsa-miR-30c-2-3p	2.24
hsa-miR-1180-3p	2.25
hsa-miR-4638-3p	2.28
hsa-miR-1306-3p	2.28
hsa-miR-5193	2.28
hsa-miR-485-3p	2.28
hsa-miR-3127-3p	2.28
hsa-miR-4664-3p	2.28
hsa-miR-4435	2.28
hsa-miR-4757-3p	2.28
hsa-miR-619-5p	2.28
hsa-miR-224-3p	2.38
hsa-miR-7641	2.45
hsa-miR-93-5p	2.58
hsa-let-7e-3p	2.58
hsa-miR-155-5p	2.58
hsa-miR-128-1-5p	2.59
hsa-miR-125b-1-3p	2.67
hsa-miR-99b-5p	2.69
hsa-miR-21-3p	2.69

hsa-miR-125b-2-3p	2.71
hsa-miR-125b-5p	2.77
hsa-miR-642a-5p	2.77
hsa-miR-486-3p/486-5p	2.83
hsa-miR-1343-3p	2.91
hsa-miR-2277-5p	2.92
hsa-miR-212-3p	2.93
hsa-miR-18a-3p	2.93
hsa-miR-500b-5p	2.95
hsa-miR-501-3p	3.01
hsa-miR-502-3p	3.01
hsa-miR-3605-3p	3.15
hsa-miR-3200-3p	3.25
hsa-miR-425-3p	3.47
hsa-miR-501-5p	3.51
hsa-miR-210-5p	3.59
hsa-miR-193b-3p	3.71
hsa-miR-181b-5p	3.72
hsa-miR-652-3p	3.89
hsa-miR-125a-3p	3.89
hsa-miR-125a-5p	4.06
hsa-miR-455-3p	4.06
hsa-miR-532-3p	4.14
hsa-miR-27a-5p	4.28
hsa-miR-877-5p	4.30
hsa-miR-574-3p	4.38
hsa-miR-500a-3p	4.64
hsa-miR-6511a-3p	4.73
hsa-miR-874-5p	4.75
hsa-miR-106b-3p	4.79
hsa-miR-93-3p	4.83
hsa-miR-92a-1-5p	4.86
hsa-miR-99b-3p	4.95
hsa-miR-671-3p	5.00
hsa-miR-30d-5p	5.01
hsa-miR-30c-1-3p	5.06
hsa-miR-219a-1-3p	5.08
hsa-miR-149-5p	5.11
hsa-miR-744-5p	5.16
hsa-miR-331-3p	5.36
hsa-miR-339-3p	5.42
hsa-miR-574-5p	5.60
hsa-miR-1296-5p	5.63
hsa-miR-205-5p	5.64
hsa-miR-15b-5p	5.68
hsa-miR-132-3p	5.74

hsa-miR-181a-5p	5.80
hsa-miR-589-3p	5.83
hsa-miR-328-3p	5.99
hsa-miR-1303	6.02
hsa-miR-324-5p	6.02
hsa-miR-6842-3p	6.03
hsa-miR-222-3p	6.11
hsa-miR-31-5p	6.37
hsa-miR-197-3p	6.40
hsa-miR-361-3p	6.54
hsa-miR-505-3p	6.62
hsa-miR-92a-3p	6.65
hsa-miR-2110	6.75
hsa-miR-874-3p	6.88
hsa-miR-324-3p	6.99
hsa-let-7d-3p	7.03
hsa-miR-3615	7.15
hsa-miR-200b-5p	7.18
hsa-miR-330-5p	7.36
hsa-miR-484	7.44
hsa-miR-200c-3p	7.89
hsa-miR-589-5p	8.01
hsa-miR-423-5p	8.10
hsa-miR-1301-3p	8.17
hsa-miR-423-3p	8.28
hsa-miR-1307-3p	8.33
hsa-miR-146b-3p	8.34
hsa-miR-25-5p	8.57
hsa-miR-148b-3p	9.42
hsa-miR-320a/b/c/d	10.13
hsa-miR-342-5p	12.51
hsa-miR-887-3p	12.90
hsa-miR-7706	13.63
hsa-miR-130b-5p	13.96
hsa-miR-941	14.01

A negative SAM score indicates an increase in expression when treated with eCig liquid. A positive SAM score indicates a decrease in expression when treated with eCig liquid.

Supplemental Table 2: SAM-Seq analysis (at median FDR=0) identified 125 miRNA as significantly differentially expressed between cells treated with any vaporized liquid (n=6) compared to any non-vaporized liquid (n=6).

miRNA	Observed SAM score(d)
hsa-miR-7706	-4.19
hsa-miR-484	-4.00
hsa-miR-330-3p	-3.52
hsa-miR-874-3p	-3.19
hsa-miR-4709-3p	-2.93
hsa-miR-1306-5p	-2.84
hsa-miR-339-3p	-2.80
hsa-miR-6511a-3p	-2.77
hsa-miR-1268b	-2.77
hsa-miR-6511b-3p	-2.73
hsa-miR-146b-3p	-2.62
hsa-miR-887-5p	-2.61
hsa-miR-219a-1-3p	-2.56
hsa-miR-125b-2-3p	-2.56
hsa-miR-744-5p	-2.54
hsa-miR-5001-3p	-2.49
hsa-miR-361-3p	-2.48
hsa-miR-3909	-2.43
hsa-miR-887-3p	-2.38
hsa-miR-4645-3p	-2.34
hsa-miR-542-5p	-2.33
hsa-miR-3615	-2.29
hsa-miR-1249-3p	-2.15
hsa-miR-3929	-2.04
hsa-miR-1285-3p	-2.00
hsa-miR-4449	-1.99
hsa-miR-548au-5p/548c-5p/548o-5p/548am-5p	-1.99
hsa-miR-1260b	-1.95
hsa-miR-200b-5p	-1.93
hsa-miR-1301-3p	-1.92
hsa-miR-1247-5p	-1.80
hsa-miR-25-5p	-1.78
hsa-miR-2277-5p	-1.69
hsa-miR-455-3p	-1.67
hsa-miR-1254	-1.59
hsa-miR-6724-5p	-1.57
hsa-miR-6510-3p	-1.57
hsa-let-7d-3p	-1.52
hsa-miR-1229-3p	-1.52
hsa-miR-1343-3p	-1.52

hsa-miR-130b-5p	-1.46
hsa-miR-15b-5p	-1.45
hsa-miR-1266-5p	-1.43
hsa-miR-877-5p	-1.43
hsa-miR-4758-3p	-1.42
hsa-miR-331-3p	-1.42
hsa-miR-29b-2-5p	-1.39
hsa-miR-941	-1.38
hsa-miR-1296-5p	-1.35
hsa-miR-500a-3p	-1.35
hsa-miR-99b-3p	-1.34
hsa-let-7c-3p	-1.29
hsa-miR-1304-3p	-1.25
hsa-miR-6499-5p	-1.21
hsa-miR-125a-3p	-1.19
hsa-let-7a-3p	-1.17
hsa-miR-449c-3p	-1.14
hsa-miR-378a-5p	-1.13
hsa-miR-1307-3p	-1.13
hsa-miR-27b-5p	-1.13
hsa-miR-3605-5p	-1.13
hsa-miR-501-3p	-1.13
hsa-miR-589-3p	-1.12
hsa-miR-23a-5p	-1.08
hsa-miR-155-5p	-1.08
hsa-miR-210-5p	-1.06
hsa-miR-3174	-1.00
hsa-miR-1260a	-1.00
hsa-let-7f-2-3p	-1.00
hsa-miR-4797-3p	-1.00
hsa-miR-3605-3p	-0.98
hsa-miR-6810-3p	-0.98
hsa-miR-6769b-3p	-0.97
hsa-miR-210-3p	-0.95
hsa-miR-671-3p	-0.87
hsa-miR-212-5p	-0.87
hsa-miR-132-3p	-0.85
hsa-miR-221-5p	-0.83
hsa-miR-193a-5p	-0.83
hsa-miR-3166	-0.83
hsa-miR-181a-2-3p	-0.80
hsa-miR-30c-2-3p	-0.78
hsa-miR-548i	-0.76
hsa-miR-3928-3p	-0.71
hsa-miR-1180-3p	-0.70
hsa-miR-1303	-0.68

hsa-miR-769-5p	-0.66
hsa-miR-92b-5p	-0.65
hsa-miR-708-3p	-0.64
hsa-miR-708-5p	-0.61
hsa-miR-374b-5p	-0.60
hsa-miR-449b-3p	-0.59
hsa-miR-15a-5p	2.29
hsa-miR-130a-3p	2.33
hsa-miR-424-5p	2.37
hsa-miR-660-5p	2.39
hsa-miR-7-5p	2.43
hsa-miR-101-3p	2.57
hsa-miR-330-5p	2.58
hsa-miR-141-5p	2.62
hsa-miR-374a-5p	2.64
hsa-miR-29c-5p	2.67
hsa-miR-450b-5p	2.70
hsa-miR-223-3p	2.74
hsa-miR-135b-3p	2.80
hsa-miR-30d-3p	2.82
hsa-miR-126-3p	2.91
hsa-miR-3912-3p	3.00
hsa-miR-22-5p	3.04
hsa-miR-29b-3p	3.08
hsa-miR-126-5p	3.19
hsa-let-7i-3p	3.24
hsa-miR-106b-5p	3.32
hsa-miR-7-1-3p	3.38
hsa-miR-23c	3.39
hsa-miR-561-5p	3.65
hsa-miR-335-5p	3.69
hsa-miR-4454	4.01
hsa-miR-29c-3p	4.03
hsa-miR-455-5p	4.15
hsa-miR-548av-5p/548k	4.35
hsa-miR-135b-5p	4.36
hsa-miR-194-5p	4.60
hsa-miR-181a-3p	5.65
hsa-miR-450a-5p	6.19

A negative SAM score indicates an increase in expression when treated with vaporized eCig liquid. A positive SAM score indicates a decrease in expression when treated with vaporized eCig liquid.
